# Innovative Approach to Producing Palladium-103 for Auger-Emitting Radionuclide Therapy: A Proof-of-Concept Study

**DOI:** 10.3390/ph17020253

**Published:** 2024-02-16

**Authors:** Aicha Nour Laouameria, Mátyás Hunyadi, Attila Csík, Zoltán Szűcs

**Affiliations:** 1 Doctoral School of Chemistry, University of Debrecen, Egyetem tér 1, H-4032 Debrecen, Hungary; 2 HUN-REN Institute for Nuclear Research, Bem tér 18/c, 4026 Debrecen, Hungary; hunyadi.matyas@atomki.hu (M.H.); csik.attila@atomki.hu (A.C.); zszucs@atomki.hu (Z.S.)

**Keywords:** Auger electron therapy, palladium-103, partial pressure, radioisotope separation, diffusion

## Abstract

Auger-emitting radionuclides, exemplified by Pd-103, exhibit considerable therapeutic potential in cancer treatment due to their high cytotoxicity and localized biological impact. Despite these advantages, the separation of such radionuclides presents a complicated challenge, requiring intricate and time-intensive “wet chemistry” methods attributed to the exceptional chemical inertness of the associated metals. This study proposes an innovative solution to this separation challenge through the design and implementation of a piece of radionuclide separation equipment (RSE). The equipment employs a dry distillation approach, capitalizing on differences in partial vapor pressures between irradiated and resulting radioactive metals, with a diffusion-driven extraction method applied to separate Pd-103 radionuclides generated via the proton irradiation of Rh-103 at cyclotron. Our optimization endeavors focused on determining the optimal temperature for effective metal separation and adjusting the diffusion, evaporation, and deposition rates, as well as addressing chemical impurities. The calculations indicate 17% ± 2% separation efficiency with our RSE. Approximately 77 ± 2% and 49 ± 2% of the deposited Pd-103 were isolated on substrates of Nb foil and ZnO-covered W disc, respectively. The proposed innovative dry distillation method that has been experimentally tested offers a promising alternative to conventional separation techniques, enabling enhanced purity and cost-efficient cancer treatment strategies.

## 1. Introduction

Auger-emitting radionuclides have potential in therapy for cancer due to their high level of cytotoxicity and short-range biological effectiveness [1]. As a result of the decay processes of these radionuclides, a series of low-energy electrons are ejected in what is referred to as the Auger effect [2], which has already been explored as a potential source for targeted radiotherapy. The Auger effect is based on the emission of an electron following various excitation processes, such as electron capture (EC), internal conversion (IC), or photoelectric X-ray absorption [3]. The unique properties of Auger electrons make them an attractive choice for pinpoint accuracy in internal radiotherapy. Auger electrons have a very short range, typically less than the size of a cell, due to their low energy with respect to β-electrons [4]. This makes them capable of inducing double-stranded DNA breaks [5], a critical mechanism in the context of their therapeutic impact. Further, the short ranges of Auger electrons of less than a cell diameter make it theoretically possible to effectively irradiate targeted cells while largely sparing the surrounding healthy tissues. For these reasons, Auger radiotherapy is considered a promising emerging field in nuclear medicine, especially for targeting very small tumor masses, such as metastases.

**Figure 1:**
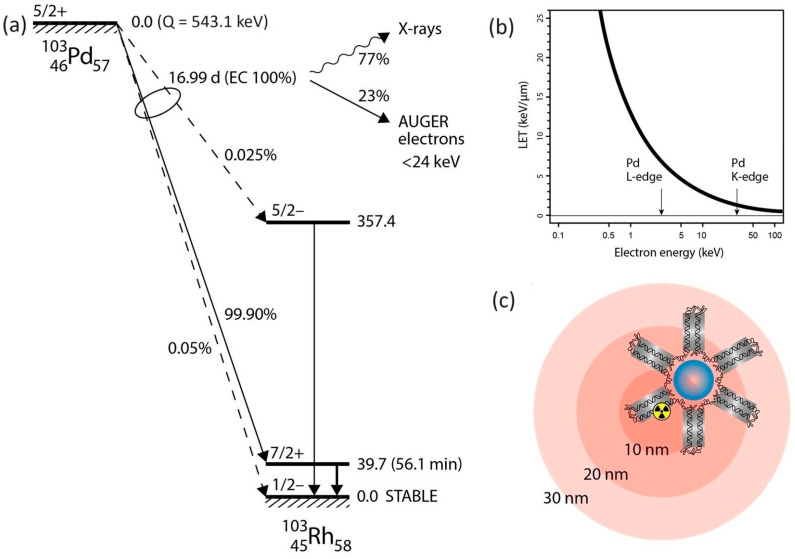
(**a**) Decay scheme of Pd-103 showing the subsequent decay of core–hole states via characteristic X-ray and Auger electrons. (**b**) Stopping power of electrons in an exemplary organic medium (guanine) for the energy range of typical Auger electrons calculated via the ESTAR code [9]. (**c**) Illustration of impact range of Auger electrons compared to the dimensions of chromatin strands.

Palladium-103 (Pd-103) decays to rhodium-103 m (Rh-103 m) via electron capture (EC) with a half-life of 16.99 days (Figure 1a). This process leaves a core–hole state that recombines via deexcitation cascades emitting characteristic X-rays (K-edge at 24.37 keV) and Auger electrons with a branching ratio of 77% and 23%, respectively [6]. From a technical point of view, the relatively low energy of X-ray photons allows for the non-problematic shielding, handling, storage, and elution of a Pd-103-based generator [7]. Furthermore, Pd-103 already enjoys widespread medical use by virtue of its X-ray emissions, utilized in brachytherapy [8].

In Auger transitions, a cascade process is generated via the deexcitation of the initial deep hole state. During this process, numerous low-energy electrons are emitted with a few hundred eV, which have a steeply increasing stopping power (linear energy transfer–LET) with decreasing energy (Figure 1b). These low-energy electrons have a stopping range of 30–50 nm, comparable with the average diameter of chromatin strands (~30 nm), as illustrated in Figure 1c.

The production of Pd-103 from stable rhodium-103 (Rh-103) using a cyclotron has been reported, mainly through the ^103^Rh(p,n)^103^Pd reaction [10,11]. Furthermore, an Auger electron emitter should be produced via a low-cost process while maximizing specific activity and purity to present therapeutic potential [12,13]. However, the separation process of the Pd-103 from the target material remains challenging and is usually carried out via wet chemistry [14], which is a very complicated and laborious process due to the extreme chemical inertness of these elements. Additionally, it involves the use of hazardous chemicals, resulting in a lower rhodium recovery rate and the generation of radioactive waste [15], considering that the cost of rhodium material proves excessively expensive for any chemical processing.

**Figure 2:**
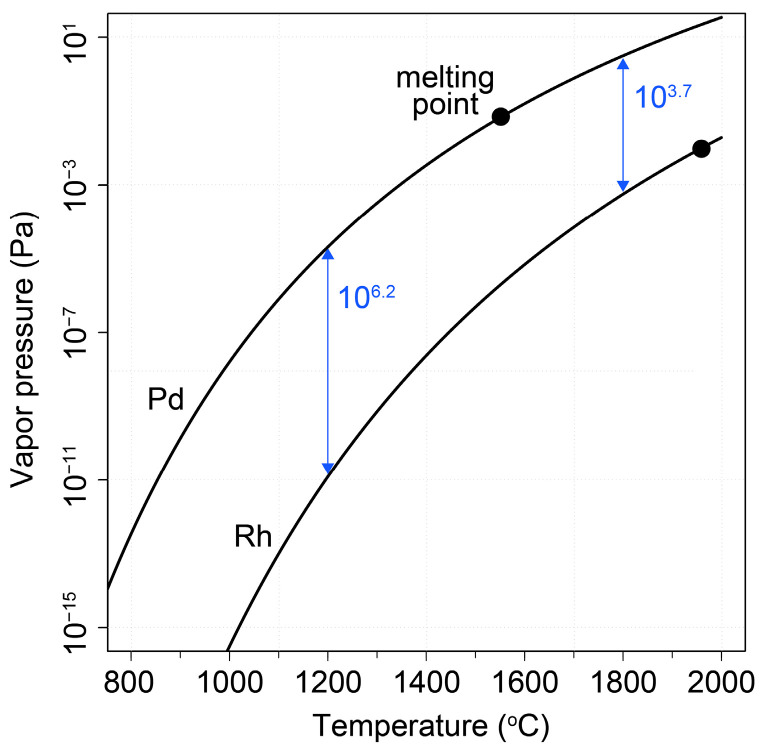
Partial vapor pressure of palladium and rhodium as a function of the temperature [17].

In this study, we propose a more feasible method to address this challenge by employing diffusion-driven extraction for the separation of the Pd-103 radionuclide generated by irradiating a natural Rh-103 target with protons at the cyclotron facility of HUN-REN Institute for Nuclear Research (ATOMKI). We have designed and constructed radionuclide separation equipment (RSE) based on the dry distillation method, which relies on the differences in partial vapor pressures between the target and the resulting radioactive materials. In practice, this principle of operation only holds for the case when the radionuclide material produced via irradiation has at least two orders of magnitude higher vapor pressure than the target material to reach a separation factor of at least 100. The vapor-pressure functions of the palladium–rhodium couple are compared and apparently obey this criterion (Figure 2). The experimental validation of the proof-of-principle and the determination of operational characteristics of the expected new separation method via thermo-diffusion [16] have been conducted. Additionally, a technical design is proposed by calculating thermally driven processes of atomic diffusion, selective evaporation, and deposition of palladium.

## 2. Results

### 2.1. Characterization of Elemental Interdiffusion at the Palladium–Rhodium Interface

The most critical factors that determine the feasibility of the dry distillation technique are the advantageous relative vapor pressure of palladium and rhodium, and the diffusion coefficient of palladium in the rhodium matrix. The latter quantity was examined in specific multi-layer samples comprising a palladium–rhodium interface, which were annealed at different temperatures from 600 to 900 °C. The interdiffusion rates in the annealed samples were analyzed via depth profiling measurements using the secondary neutral mass spectrometry (SNMS) technique. The equipment was operated in direct bombardment mode by using Ar+ ions with a low energy for sputtering (E = 350 eV) and with a current density of ~1 mA/cm^2^. The erosion area was confined to a circle of 2 mm in diameter by means of a Ta mask. The lateral homogeneity of the ion bombardment was checked by a profilometer (Ambios Technology Inc., USA, Santa Cruz, CA, Model: XP-I) to analyze the depth of the crater sputtered and to convert the sputtering time to depth scale.

**Figure 3:**
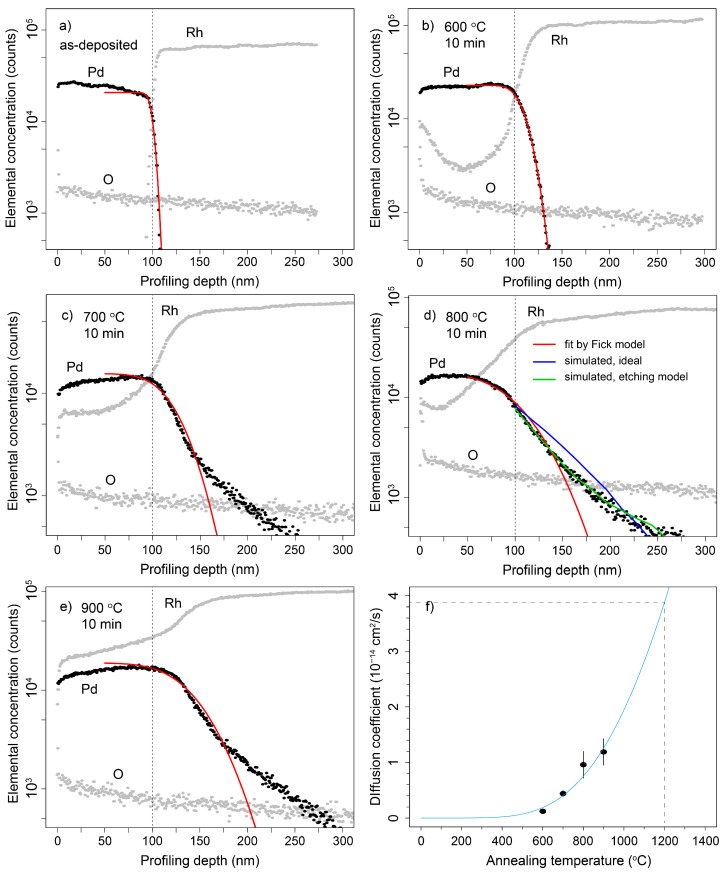
SNMS depth profiling analysis for: (**a**) the control sample, (**b**) the annealed sample at 600 °C for 10 min, (**c**) the annealed sample at 700 °C for 10 min, (**d**) the annealed sample at 800 °C for 10 min, and (**e**) the annealed sample at 900 °C for 10 min. (**f**) Palladium-in-rhodium diffusion coefficient deduced by fitting palladium profiles is plotted as a function of the annealing temperature. The fit (blue curve) results in an activation energy of palladium displacement of 0.55 eV. The extrapolated value of ~4 × 10^−14^ cm^2^/s is calculated for 1200 °C, the temperature at which the separation experiment was carried out.

Our findings revealed a noteworthy degree of elemental interdiffusion between the two constituents, as shown in Figure 3, yielding valuable insights for the formulation of subsequent systematic measurements. The obtained depth profile exhibited characteristics indicative of an ideal depth profile; however, at more advanced stages of the diffusion process (>700 °C), a long tail emerges that can be attributed to the effect of surface roughness. Indeed, the etching speed of Ar+ ions may sensitively depend on the surface morphology, referring to the common experience, that peaks are eroded faster than valleys, eventually leading to a distorted reconstruction of depth profiles. An ab initio simulation of the etching process has been carried out. We assumed a model function for the etching speed describing its dependence on the local morphology of the surface, aligning with common observations that peaks undergo erosion at a faster rate than valleys. Figure 3d illustrates the least-square fit of the model (green curve), which is compared to the depth profile of uniform etching (blue curve) with the same diffusion coefficient used as a single parameter. The surface quality may critically affect the accuracy of the deduced diffusion coefficient.

Upon annealing at 600 °C, a discernible accumulation of rhodium at the surface was evident, suggesting the plausibility of rhodium diffusion through the palladium layer via a grain boundary diffusion mechanism [18], which occurred at a faster rate than the diffusion of palladium into the rhodium foil. Subsequent annealing at higher temperatures further substantiated this phenomenon, revealing a temperature-dependent increase in rhodium content within the palladium layer. Ultimately, this led to the attainment of a palladium layer fully impregnated with rhodium, coinciding with the concurrent diffusion of palladium into the rhodium foil.

A preliminary value for the lattice diffusion constant of the palladium-in-rhodium matrix at the annealing temperatures could be obtained, yielding 0.2–1.5 × 10^−14^ cm^2^/s, by fitting the palladium concentration profiles with the formulation of Fick’s second law [19,20]:

(1) $$N=N_{0\;}\mathrm{erfc}\left(\frac{x}{2\sqrt{Dt}}\right),$$

where x is the penetration depth of palladium atoms, D is the diffusion coefficient, and t is the annealing time. By fitting the obtained values with the Arrhenius equation, an activation energy of *E_A_* = 0.55 eV was obtained:

(2) $${D=D}_{0}\left(-\frac{E_{A}}{RT}\right).$$

An approximate value of *D* = 4 × 10^−14^ cm^2^/s for the diffusion coefficient was also deduced via extrapolation to 1200 °C, which is still a moderate value that can be practically relevant only for thin layers and the minute-scale operation of the equipment. However, a systematic measurement of depth profiles over a wider temperature range and interdiffusion corrections will be needed for the accurate determination of absolute extraction rates.

### 2.2. Purity Analysis of Palladium Separation

**Figure 4:**
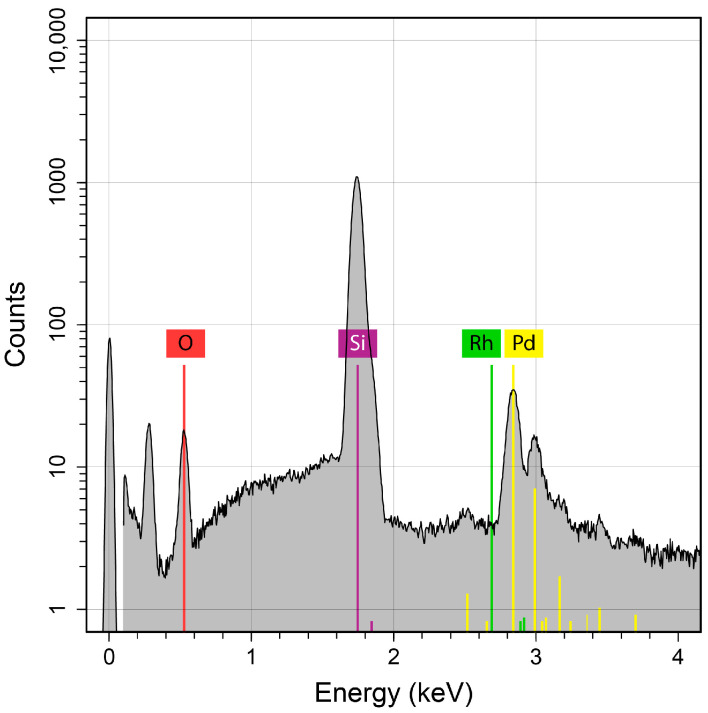
Elemental composition of the deposited layer on a silicon substrate examined via EDX (energy-dispersive X-ray spectroscopy).

To verify the separation power of the dry distillation method proposed for Pd-103, an evaporation probe was performed, with a powder mixture of natural (inactive) palladium and rhodium with a 1:1 stoichiometric ratio. The substrate used for probing the deposition was a silicon wafer of 1 mm thickness. The evaporation test was performed at 1200 °C for 10 min. The compositional purity of the deposited palladium layer was examined via scanning electron microscopy (SEM) using energy-dispersive X-ray spectroscopy (EDX). The analysis of the EDX spectrum resulted in a Rh decontamination factor greater than 100, estimated from the observed absence of rhodium content at the expected location, considering the detection limit of 0.1 at% specified for the spectrometer (Figure 4).

The initial mass of the powder mixture was measured as 29 mg, while, after the heating process, the remaining material was 23 mg. Assuming a negligible evaporation rate for the rhodium, the evaporated palladium was calculated to be 6 mg, indicating that the separation power and material purity are satisfactory even at a moderate operational temperature (1200 °C).

### 2.3. Quantitative Analysis of Pd-103 Using γ-Spectroscopy

The quantification of Pd-103 activity was performed with the utilization of a high-purity germanium (HPGe) detector. The specimens that were subjected to measurement included the rhodium foil after each irradiation and separation test, the niobium substrate post separation test and subsequent treatments with hydrochloric acid, the ZnO-covered W disc after the separation test and subsequent treatments with hydrochloric acid and nitric acid, and the rinsed liquid containing the Pd-103 isolated in vials post each dissolution. Gamma peak areas were efficiently corrected, and the Pd-103 activity of the samples was determined from the following well-known γ line of Pd-103 at 357.45 keV with a low branching ratio of 0.0221% [21]. The measurement yielded an end-of-bombardment (EOB) activity of 31.9 MBq for the separation test with the W disc.

### 2.4. Separation Experiments: Evaporation and Recovery Efficiency of Pd-103

**Figure 5:**
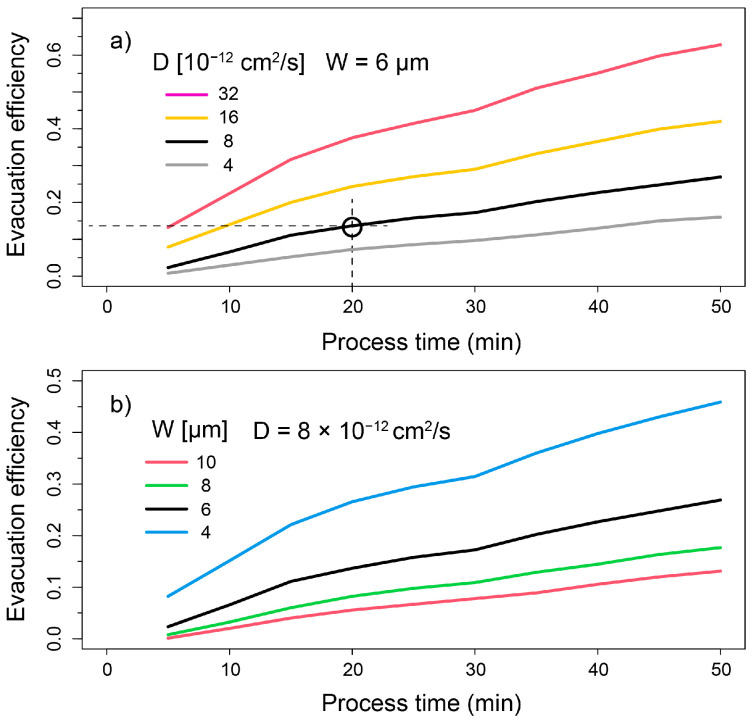
Simulated evacuation efficiency as the ratio of emitted Pd-103 with respect to the total amount in the rhodium foil. The evacuation efficiency is plotted at varying (**a**) diffusion coefficients *D* and (**b**) foil thicknesses (W). The measured ratio of evaporated-to-produced Pd-103 is also provided (open circle).

The separation of Pd-103 produced via ion-beam irradiation was performed in two subsequent steps. First, the irradiated rhodium foil was heated up in the RSE to evaporate and deposit the Pd-103 onto the substrate via distillation. The evaporation efficiency of the palladium-in-rhodium matrix was also investigated with a random-walk simulation, which described the dependence of evaporation rates on the process time and film thickness, as well as the diffusion coefficient. The simulated evaporation efficiencies are plotted in Figure 5 as a function of the process time. The simulation determines the moment when particles escape from the foil, reaching either of the two surfaces. Within a specified process time, the fraction of escaped particles relative to the initial number of particles is determined and given as the term evaporation efficiency. Fitting this evaporation efficiency to the experimental value of 14% obtained in the well-controlled separation experiment using the ZnO/W disc at 20 min of annealing, we could determine a diffusion coefficient approximately two orders of magnitude higher than the one determined from the depth-profiling analysis. A potential explanation for this discrepancy may be related to the oversimplified model of the random-walk diffusion, which used a constant time step of ∆t=1 μs and a displacement element $∆x=\sqrt{2D∆t}$, Additionally, it did not account for contributions stemming from atomic migration on grain boundaries, temperature-dependent displacement energy, or the diffusion of rhodium atoms to the opposite direction.

In the separation experiment, the measured activity on the ZnO/W substrate was 25% of the missing activity in the rhodium target. Calculating the solid angle of the deposition substrate viewed by the target foil, we obtained 2.75 sr, which can be converted to a coverage of 22%. This value agrees well with the measured ratio of activity deposition.

Following the distillation process, the substrates were treated with acidic digestion to recover the deposited Pd-103 and prepared for further chemical processing. These steps were initially tested in a pilot experiment using niobium foil as the deposition substrate. The aim of the experiment was to assess the feasibility of the distillation technique and acquire relevant technical information for the equipment design. Despite the low development level of the equipment, a considerable amount of Pd-103 activity was separated without rhodium contamination. The RSE design later incorporated accurate temperature control, thermal shielding, a cooling system, improved electric contacts, and a heating cell design. The separation experiment was then repeated using a ZnO-covered W disc as the deposition substrate. The parameters and processing efficiencies obtained in the two experiments are detailed in Table 1. The ZnO layer was hypothesized to accumulate the Pd-103 and prevent its burial within the W substrate. The comparison of the two experiments apparently showed a higher separation efficiency when the Nb substrate was used; however, both cases confirm the applicability of the proposed technique.

The rinsed Pd-103, collected in vials, underwent measurement using ICP-MS to determine the total palladium content, enabling the calculation of the specific activity, which was found to be 8.1 GBq/g.

## 3. Discussion

We have systematically examined the operational parameters of the separation equipment in correlation with the extraction efficiency of Pd-103. We have concluded from the palladium/rhodium interdiffusion measurements that significant palladium extraction rates can be achieved at a lower temperature with respect to the melting point of palladium (1552 °C) at a selected value of operational temperature (1200 °C) and duration (20 min). The Pd-103 separation yielded an activity of 31 MBq measured for the γ line at 357.45 keV in Pd-103, while a slowly increasing activity from Rh-103 m (39.7 keV) was also observed. This quantity is considered notably substantial and well suited for conducting pre-clinical investigations, involving a minimum of approximately one adult rat model, as anticipated [22]. The chemical purity of the deposited layer could not be measured with the surface analytical instruments; instead, we relied on the systematic measurements performed on inactive samples, which revealed that a significant presence of rhodium was not observed in the deposited layer.

Two main concerns must be discussed to reconsider the technical steps that are essential for the maximization of the extraction efficiency. One is the thickness of the rhodium foil, which was chosen to be 6 µm in our pilot experiments. A thicker foil, around 100 µm, could be optimally adjusted to the wide cross-section peak of the ^103^Rh(p,n)^103^Pd reaction, which may result in higher-activity production batches. In contrast, the extraction rates governed by the diffusion do not favor thick layers, but long operation times might compensate for the low evaporation rates. In our case, the 6 µm-thick target was nearly suitable for producing measurable trace amounts of radioactivity with the short irradiation time of a few hours and sufficiently thin to emit a significant fraction of the produced Pd-103.

The second concern is related to the deposition efficiency in terms of adequate material selection for the deposition surface adapted to the subsequent dissolution process. During the engineering phase of our separation equipment, a pilot experiment was performed to determine the recovery efficiency as the ratio of dissolvable Pd-103 content with respect to the total amount of deposited Pd-103. Since the deposition surface is also heated up via thermal radiation from the crucible side, an elevated rate of diffusion increases the amount of buried Pd-103 that cannot be recovered even with hard acidic digestion. The deposition surface in our studies was, first, a Nb foil without any surface treatment, then was replaced with a W disc, which was covered with a ZnO layer to accumulate Pd-103 atoms and to prevent their penetration into the W disc, as well as an appropriate substrate for both high-temperature operation and acidic dissolution. As a result, 23% of the deposited Pd-103 remained on the Nb foil, while 51% diffused into the W disc. In the former case, the higher recovery ratio can be attributed to the highly polished surface of the Nb foil. In comparison with the case of the W disc, first disregarding the effect of the ZnO layer, we may conclude that the recovery ratio is highly sensitive to the material choice and surface morphology, as well as the temperature. However, this experiment also confirmed the expected role of the ZnO layer as a diffusion barrier in contrast to the apparently insufficient thickness of 100 nm. To identify the benefit of employing metal oxides as a Pd-103 accumulator layer on the deposition substrates, systematic investigations are recommended.

## 4. Materials and Methods

The rhodium foil with a thickness of 6 μm, the niobium foil with a thickness of 50 µm, the boron nitride (BN) ceramic, and the tungsten (W) metal disc were purchased from Goodfellow, UK. The silicon wafer of 1 mm thickness was purchased from Crystal GmbH, Germany. The rhodium and palladium powders were purchased from Koch-Light Laboratories Limited, UK. The zinc oxide (ZnO) layer (100 nm thick) was fabricated at ATOMKI via atomic layer deposition (ALD) technique in a Beneq TFS-200 reactor [23]. The ZnO layer was prepared by applying diethyl-zinc (DEZ) and water (H_2_O) precursors (purchased from Sigma-Aldrich) at 100 °C reactor temperature. The consecutive pulse times were 0.3 s for DEZ followed by 10 s nitrogen purge and 0.2 s for H_2_O followed by 10 s nitrogen purge. Hydrochloric acid (HCl) of 37 m/m % and nitric acid (HNO_3_) of 65 m/m % solvents were purchased from Sigma-Aldrich and utilized without further purification.

All material characterization and experiments were carried out at ATOMKI. Measurements of radioactive rhodium were conducted utilizing a high-purity germanium (HPGe) detector—Canberra type 2002 CSL, USA. The γ-spectra were analyzed with Genie-2000 software package. The deposition of palladium onto the rhodium foil was accomplished through the magnetron sputtering deposition technique using magnetron heads made by Kurt J. Lesker. The base pressure of the sputtering chamber was lower than 2 × 10^−7^ mbar. Circular palladium targets (purchased from Kurt J. Lesker) of 5 cm diameter were used as sputtering sources. During the layer deposition, the Ar (99.999%) pressure (under dynamic flow) and the sputtering power were 5 × 10^−3^ mbar and 40 W, respectively. The sputtering rate was calculated from the layer thickness measured using an AMBIOS XP-1 profilometer. Annealing procedures were carried out using a vacuum furnace at base pressure of 7.5 × 10^−7^ mbar. The depth profile analysis of the samples was performed using a secondary neutral mass spectrometer (SNMS, type INA-X, produced by SPECS GmbH) in direct bombardment mode by using Ar+ ions with low energy for sputtering (E = 350 eV) and with a current density of ~1 mA/cm^2^. Post-ionized neutral particles were analyzed using a Balzers QMA 410 quadruple mass spectrometer; the erosion area was confined to a circle of 2 mm in diameter by means of a Ta mask. Surface morphology of the as-prepared and annealed samples was performed with a dual-beam scanning electron microscope from Thermo Fisher Scientific (FIB-SEM, Waltham, MA, USA, Model: Scios 2), equipped with Bruker type Quantax Energy Dispersive X-ray system (EDS) for composition analysis. Measurements of rinsed Pd-103 samples were carried out using the ICP-MS Thermo Scientific X2, Waltham, MA, USA.

### 4.1. Engineering and Optimization of the Thermoregulation System for the RSE

**Figure 6:**
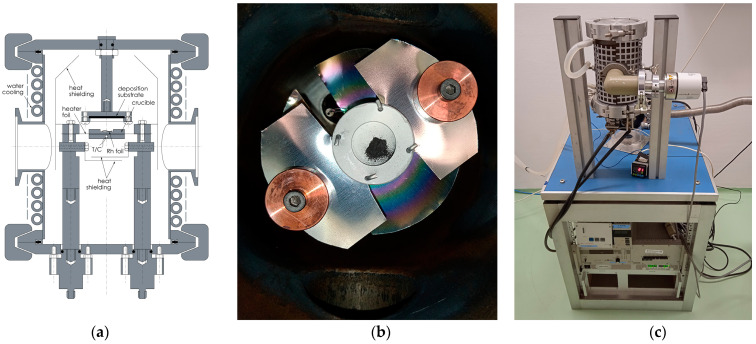
(**a**) Conceptual layout of the separation equipment incorporating a high-temperature effusion cell. (**b**) Overhead view of the BN crucible laden with a palladium/rhodium powder mixture for the purity analysis of the separation procedure. (**c**) Image of the separation equipment and control electronics.

A dedicated system was engineered and constructed, as illustrated in Figure 6. In the pursuit of isolating Pd-103 from the irradiated rhodium foil, a temperature range of 1200 to 1500 °C was selected for the pilot experiments, while, for the separation experiment, we set 1200 °C and 1–3 × 10^−5^ mbar pressure, considering the theoretically optimal conditions for the temperature and vacuum, governed by the vapor pressure curves of the two metals (Figure 2).

The RSE operation is based on resistive heating using a 50 μm-thick Nb foil cut into a specific shape to concentrate heat dissipation on the BN crucible (Figure 6). The temperature of the crucible was measured with a D-type thermocouple (Re 3%/W, Re 25%/W) that was fixed in a hole drilled in the BN block. The heating power was generated with TKD-Lambda (Genesys series 180 A/8 V) power supply.

The deposition stage comprised the substrate and mechanical base for fixing the substrate at a constant distance of 10 mm from the rhodium foil. In the pilot experiment, the substrate was a 50 μm-thick Nb foil, and in the separation experiment, a tungsten disc connected to a large copper block that was fixed to the top lid of the vacuum chamber, serving as a passive heat reservoir. For the short duration of the RSE operation (20 min), this cooling solution was found sufficient.

### 4.2. Production of Pd-103 via the Proton Irradiation of Rh-103

**Figure 7:**
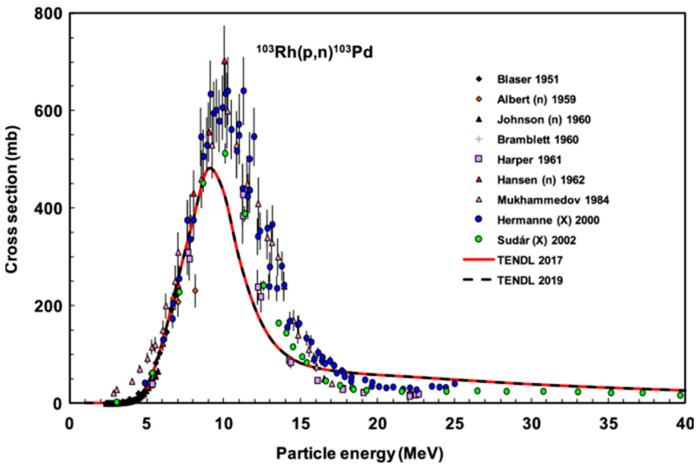
Comprehensive presentation of experimental data and TENDL predictions for the ^103^Rh(p,n)^103^Pd reaction [25] (with permission).

The ^103^Rh(p,n)^103^Pd nuclear reaction was induced with a proton beam utilizing the MGC-20 cyclotron at ATOMKI. An optimal beam energy of 10 MeV and an intensity of 20 μA were selected for this purpose. To achieve the desired activity of 10 MBq for each experimental iteration, the duration of irradiation in preliminary trials was meticulously determined by considering parameters such as the rhodium foil thickness and cross-sectional data, as illustrated in Figure 7. The duration of the first irradiation was 2 h using a proton beam energy of 14.5 MeV and an intensity of 10 μA, the second irradiation was extended to 5.5 h using a beam energy of 10 MeV and an intensity of 10 μA, and the third irradiation was further extended to 8 h using a beam energy of 11 MeV and an intensity of 20 μA.

This optimization of irradiation parameters was able to enrich 31 MBq of Pd-103 decay-corrected to the end of bombardment (EOB) during the third irradiation. The selected proton beam’s energy was deliberately kept optimally low to avoid side reactions such as ^103^Rn(p,pn)^102m,102^Rh and ^103^Rh(p,p2n)^101m,101^Rh, while ensuring sufficient induction of a usable quantity of Pd-103. The ATOMKI cyclotron’s vertical beam line system, employing helium gas for solid target cooling on the front side and water cooling on the back side, was employed for isotope production [24]. The rhodium target foil, with a thickness of 6 µm, was irradiated using a copper target holder.

### 4.3. Synthesis and Diffusivity Characterization of Palladium/Rhodium Alloy Samples

To predict the intrinsic diffusion coefficient of palladium-in-rhodium matrix, multi-layer specimens were prepared and annealed at various temperatures. Rhodium foils of 6 μm thick were covered with a 100 nm-thick layer of natural palladium employing a magnetron-sputtering vapor deposition technique. The samples were systematically annealed under high-vacuum conditions in a general-use vacuum furnace at 600 °C, 700 °C, 800 °C, and 900 °C, each for 10 min. The as-prepared and annealed samples were analyzed with SNMS depth profiling.

### 4.4. Separation of Pd-103 from Irradiated Rh-103: A Process of Isolation

Pilot experiments focused on the optimization of technical parameters for the RSE design. This included fine-tuning of electrical resistance, thermal conductance of electrode components, and calibration of the heating power in correlation with the temperature. Typically, 400 W heating power led to stable regulation at 1200 °C. Based on the output of these experimental tests, some functional elements of the equipment had to be redesigned, which included thermal radiation shielding, cooling system, thermocouple positioning, and material choice (BN) for the crucible to avoid metallic contacts and potential alloying with the rhodium foil, as well as improvement of electrode contacts. The subsequent phase involved probing the ideal temperature conditions for the efficient isolation of the two metallic elements to avoid the rhodium contamination in the deposition material. A test material consisting of equimolar masses of rhodium and palladium powders was loaded into the crucible of the RSE, as illustrated in Figure 6b. This composite was heated up to 1200 °C for 10 min. The EDX analysis resulted in a significant concentration of palladium, while that of rhodium was negligible.

Following the comprehensive optimization of parameters and the identification of the optimal temperature range for effective separation, the separation equipment was employed to execute dual separations of Pd-103 from irradiated Rh-103. These separation processes occurred after both the second and third irradiation events.

### 4.5. Pd-103 Retrieval from Deposition Surfaces through Enhanced Recovery Processes

Two distinct substrates were employed as deposition surfaces for Pd-103, isolated using our separation equipment, as part of an optimization investigation. First, in a pilot experiment, a 50 µm-thick Nb foil served as the deposition substrate for evaporated Pd-103. The Nb foil, following the evaporation test, was treated with HCl acid to harvest the deposited Pd-103. Varied volumes of HCl were applied for different durations. Subsequently, the rinsed material was collected in vials and subjected to quantitative analysis.

Following the initial pilot experiment involving Nb foil, the subsequent experiment was dedicated to improving the recovery efficiency of Pd-103. Therefore, a tungsten disc of 2 mm thickness and 30 mm diameter was employed as the deposition substrate and covered with a 100 nm-thick ZnO layer to accumulate Pd-103 atoms. We selected ZnO to serve as a diffusion barrier to minimize the penetration of Pd-103 into the W substrate, which simultaneously has a high melting point and good solubility by HCl. The W disc substrate was treated in subsequent stages of acidic digestion with HCl and HNO_3_, utilizing the same methodology as previously described, and was subsequently prepared for quantitative measurements.

## 5. Conclusions

The outcomes of our investigation have instigated a pioneering concept: the exploitation of diffusion-driven extraction to isolate radionuclides, stemming from the irradiation of Rh-103 at the cyclotron. This innovative approach presents itself as a credible alternative to conventional wet chemistry techniques for the separation of Pd-103. Following the successful separation of Pd-103 from the bulk irradiated rhodium material, a challenge emerged pertaining to the collection of the evaporated radioisotope and the need to avoid or minimize alloy formation between the trapped Pd-103 and the substrate. Subsequent optimization studies will be conducted in the future to address and resolve this challenge.

## Figures and Tables

**Table 1:** Measured activities and processing efficiency in Pd-103 separation.

	Substrate	T_evap_	∆t_evap_	EOB	Evaporated		Deposited		Recovery	
		°C	min	MBq	MBq	%_EOB_	MBq	%_evap_	kBq	%_evap_
Pilot experiment	Nb foil	~1500	10	7.87	3.54	45	1.49	42	1150	77
Separation experiment	ZnO/W disc	1200	20	31.9	4.47	14	1.12	25	547	49

## Data Availability

Data is contained within the article.
